# Comparing intra-observer variation and external variations of a fully automated cephalometric analysis with a cascade convolutional neural net

**DOI:** 10.1038/s41598-021-87261-4

**Published:** 2021-04-12

**Authors:** In-Hwan Kim, Young-Gon Kim, Sungchul Kim, Jae-Woo Park, Namkug Kim

**Affiliations:** 1 Department of Biomedical Engineering, Asan Medical Institute of Convergence Science and Technology, Asan Medical Center, University of Ulsan, College of Medicine, Seoul, 05505 Republic of Korea; 2 Transdisciplinary Department of Medicine and Advanced Technology, Seoul National University Hospital, Seoul, 03080 Republic of Korea; 3 Department of Orthodontics, Kooalldam Dental Hospital, 1418 Kyongwondaero Bupyong-Gu, Incheon, 21404 Republic of Korea; 4 Department of Radiology, Asan Medical Center, University of Ulsan College of Medicine, Seoul, 05505 Republic of Korea; 5 Department of Convergence Medicine, Asan Medical Institute of Convergence Science and Technology, University of Ulsan College of Medicine, Asan Medical Center, 88 Olympic-Ro 43-Gil Songpa-Gu, Seoul, 05505 Republic of Korea

**Keywords:** Preclinical research, Oral anatomy

## Abstract

The quality of cephalometric analysis depends on the accuracy of the delineating landmarks in orthodontic and maxillofacial surgery. Due to the extensive number of landmarks, each analysis costs orthodontists considerable time per patient, leading to fatigue and inter- and intra-observer variabilities. Therefore, we proposed a fully automated cephalometry analysis with a cascade convolutional neural net (CNN). One thousand cephalometric x-ray images (2 k × 3 k) pixel were used. The dataset was split into training, validation, and test sets as 8:1:1. The 43 landmarks from each image were identified by an expert orthodontist. To evaluate intra-observer variabilities, 28 images from the dataset were randomly selected and measured again by the same orthodontist. To improve accuracy, a cascade CNN consisting of two steps was used for transfer learning. In the first step, the regions of interest (ROIs) were predicted by RetinaNet. In the second step, U-Net detected the precise landmarks in the ROIs. The average error of ROI detection alone was 1.55 ± 2.17 mm. The model with the cascade CNN showed an average error of 0.79 ± 0.91 mm (paired t-test, p = 0.0015). The orthodontist’s average error of reproducibility was 0.80 ± 0.79 mm. An accurate and fully automated cephalometric analysis was successfully developed and evaluated.

## Introduction

Cephalometric analysis is an essential tool of orthodontic diagnosis as well as treatment planning in orthognathic surgery. The first step of cephalometric analysis requires identifying cephalometric landmarks, a labour-intensive and time-consuming task for even well-trained orthodontists. In addition, cephalometric analysis suffers from two types of errors—including projection error caused by projected X-ray images from 3D objects—and identification errors caused by incorrect identification of landmarks, tracing, and measurements^1–3^. Among these errors, the inconsistency in landmark identification may prove greater than other errors^4^. The variation of landmark definition, bony complexity of the related region, and the quality of the X-ray image could affect accuracy of landmark identification. Even after expert orthodontists received standardized training for landmark identification, disagreement between inter-observers was inevitable^5^. To overcome these problems, several studies developed automated cephalometric analysis to reduce analysis time and improve the accuracy of landmark identification^6,7^. Furthermore, various approaches to automate landmark identification have been proposed; however, these approaches have not proved accurate enough for clinical use^8–10^.

Recently, deep learning with convolutional neural networks (CNN) has shown surprising accomplishments in computer vision tasks, which can be applied to classification, detection, and semantic segmentation in medical imaging^11^. Therefore, automated landmark prediction studies have been rapidly applied to cephalometric analysis^12–15^. In order to improve the prediction performance within the search area, various image analysis methods have been proposed to preprocess the images first to find the regions of interest (ROI)^16^. Similarly, for the landmark detection, a paper for predicting a landmark after detecting an ROI has also been proposed^17^.

All these studies insisted that the automatic landmark identification system performed not only accurately, but also quickly. However, the landmark prediction error within 2 mm reported in these studies may be too large to use in a clinical situation. Some investigators divided the cephalometric X-ray image into small ROIs to increase the accuracy of automatic identification^14,18^. In addition, Hwang et al., compared the human and automated landmark identification prediction error and reported that the automated system shows more accurate results^19^. However, the accuracy of the automated landmark prediction system was only comparable to those of different users due to inter-observer variability and inferior to those of multiple trials of single user.

In this study, we proposed a cascade network to detect the related ROI of each landmark with region proposal network and find the exact position of a landmark in the ROI with semantic segmentation network—like orthodontists when determining the cephalometric landmarks—which could improve the robustness of landmark identification to the orthodontist-level.

## Materials and methods

### Dataset

This retrospective study was conducted according to the principles of the Declaration of Helsinki, and was performed in accordance with current scientific guidelines. The study protocol was approved by the Institutional Review Board Committee of Seoul National University School of Dentistry and Seoul National University Dental Hospital, Seoul, Korea (S-D 2018010 and ERI 19007). The requirement for informed patient consent was waived by the Institutional Review Board Committee of Seoul National University School of Dentistry and Seoul National University Dental Hospital. A total of 1000 consecutive lateral cephalometric X-ray images were acquired from 509 patients from the department of orthodontics in Kooalldam dental hospital from 2017 to 2018. All patients had permanent dentition without dentofacial deformity. Radiographs (n = 140) were from 140 patients who wanted to start orthodontic treatment, and the other 860 radiographs were from 369 patients who completed treatment. Although we received informed consent from all the patients, all personal information was deleted. All cephalometric X-ray images are grayscale images with the 2 k × 3 k pixel and 8-bit depth, stored in Digital Imaging and Communications in Medicine (DICOM) file format. Considering the ratio of the original image size, all cephalometric X-ray images were resized to 700 × 1000, and pixel normalization was performed by dividing by 255.0 to have pixel values in the range 0–1.

### Landmark definition

**Figure 1:**
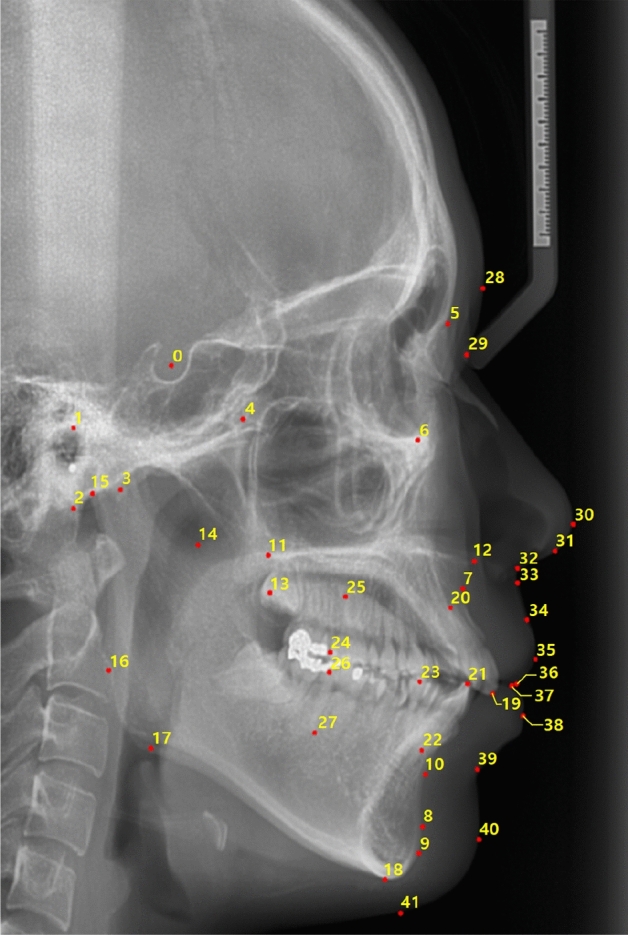
An example case of forty-two landmarks (numbered 0–41) in a cephalometric X-ray lateral image of size 2 k × 3 k pixel used in this study.

All the images were traced by one orthodontist (JWP) with more than 20 years of clinical experience. Forty-two landmarks were traced as shown in Fig. 1 and Table 1. Among them, 28 and 14 landmarks were selected from the hard tissue and soft tissue, respectively. To evaluate intra-observer variabilities, twenty-eight images from the dataset were randomly selected and measured again by the same orthodontist (JWP).

**Table 1:** The landmark names with corresponding numbers in Fig. 1.

Index	Landmark	Index	Landmark
0	Sella	21	Mandible 1 crown
1	Porion	22	Mandible 1 root
2	Basion	23	Occlusal plane point
3	Hinge axis	24	Maxilla 6 distal
4	Pterygoid	25	Maxilla 6 root
5	Nasion	26	Mandible 6 distal
6	Orbitale	27	Mandible 6 root
7	A-Point	28	Glabella
8	Protuberance menti	29	Soft tissue nasion
9	Pogonion	30	Pronasale
10	B-Point	31	Columella
11	Posterior nasal spine (PNS)	32	Subnasale
12	Anterior nasal spine (ANS)	33	Soft tissue A
13	R1	34	Labrale superius
14	R3	35	Upper lip
15	Articulare	36	Stomion superius
16	Ramus down	37	Stomion inferius
17	Corpus left	38	Lower lip
18	Menton	39	Soft tissue B
19	Maxilla 1 crown	40	Soft tissue pogonion
20	Maxilla 1 root	41	Soft tissue menton

### The cascade network

**Figure 2:**
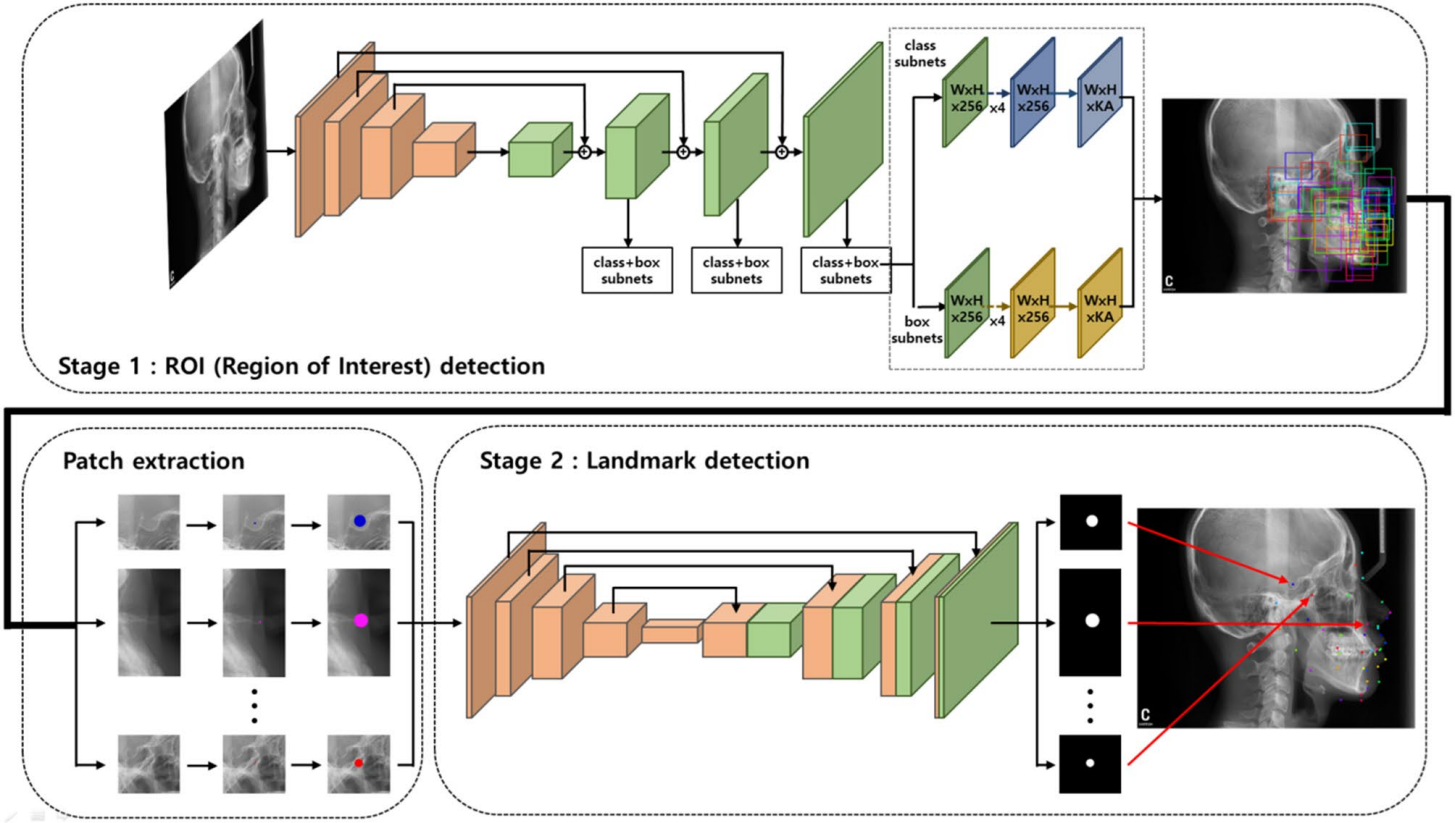
The general schematic of our proposed algorithm for finding the exact location of landmarks with a cascade network. The proposed algorithm consists of two parts, ROI detection (upper part) to propose the area of interest and the landmark prediction (lower part) to find the exact location of landmarks.

Since the cephalometric X-ray image is very large, finding the exact location of landmarks using a simple deep learning model is very challenging. To overcome this issue, we proposed a fully automated landmark prediction algorithm with a cascade network to improve prediction accuracy and reduce false-positive regions. Figure 2 shows a diagram of our proposed algorithm with the cascade network^20^. The proposed algorithm consists of two steps: (1) ROI detection and (2) landmark prediction. First, candidate ROI regions with different sizes depending on each landmark were trained by an ROI detection network. The complexity of the areas surrounding each landmark should be considered for more robust ROI detection. A different range of views is generally required when expert orthodontists identify each landmark. Applying these considerations, various ROI sizes were evaluated. Then, the exact locations of each landmark were detected based on a semantic segmentation network in the results of the previous ROI detection.

#### ROI detection

**Figure 3:**
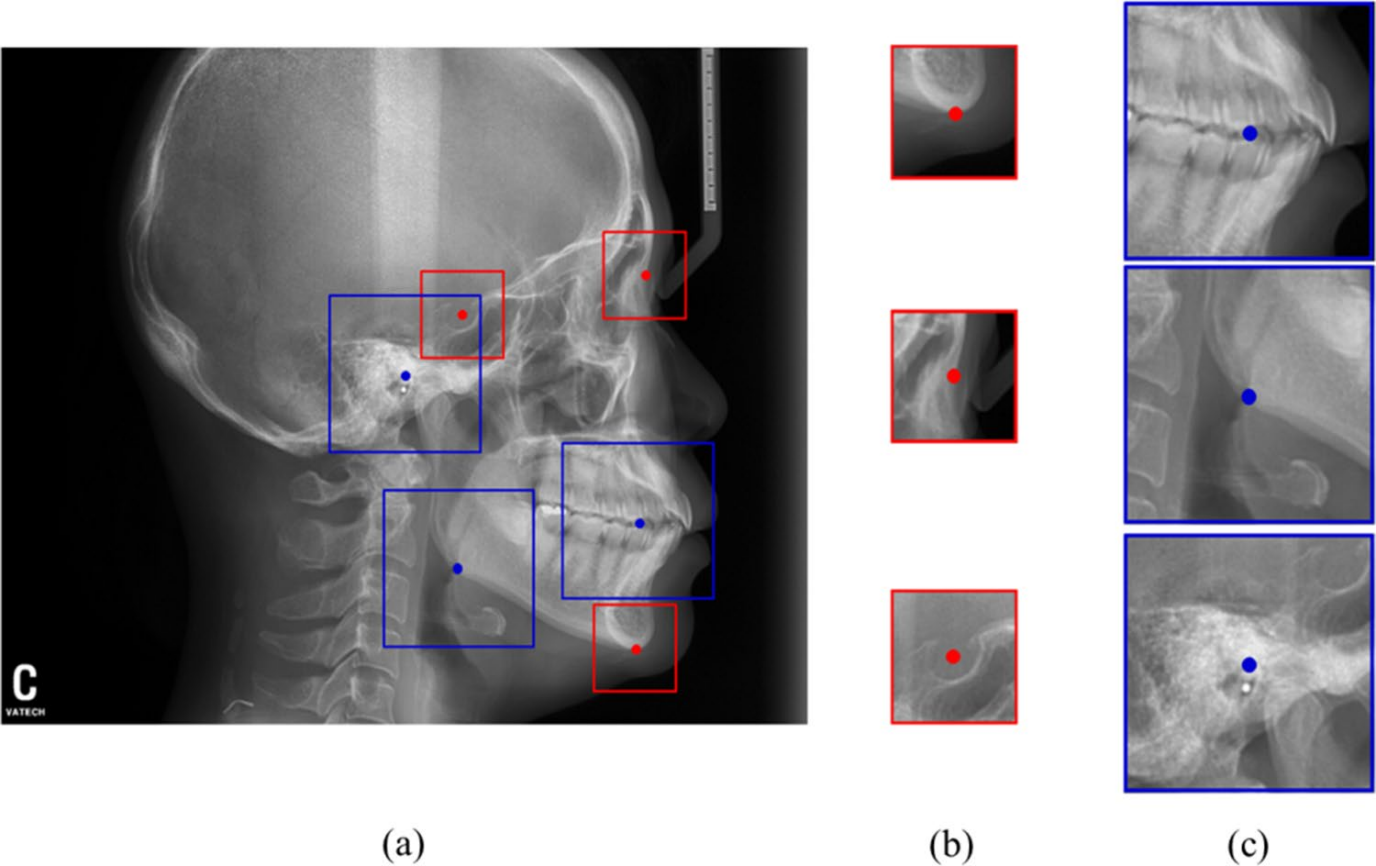
Two sizes of ROIs in the cephalometric X-ray. (**a**) ROIs with 256 × 256 and 512 × 512 size were extracted by landmarks. (**b**) Sella, nasion, and menton requiring a small ROI with 256 × 256 size (red), and (**c**) hinge, corpus and Md6 root requiring a wide ROI with 512 × 512 size (blue).

The RetinaNet, a state-of-the-art CNN based detection algorithms, was used to detect ROIs^21^. The RetinaNet is a type of one-stage detector, which selects feature pyramid network to train the model efficiently by extracting features in various sizes of the feature map. The datasets were split into training, validation, and test set at a ratio of 8:1:1. For training, the ROI patches with the centre of the landmark marked with coordinates $\left( {T_{x} , T_{y} } \right)$ were extracted. The model was trained from scratch due to relatively large dataset and preserving originality of our dataset. In Fig. 3, a different range of ROI depending on each landmark were proposed and evaluated similar to the orthodontists' viewing. The two sizes of ROI, including 256 × 256 and 512 × 512, were evaluated.

Various augmentation methods, including Gaussian noise, random brightness, blurring, random contract, flip, and random rotation, were used to train the detection model. Adam optimizer was used, Focal loss was used, and the accuracy of the ROI detection model was expressed using the Euclidean distance between the centre point $\left( {T_{x} , T_{y} } \right){ }$ and the predicted ROI patch $\left( {P_{x} , P_{y} } \right)$ from the ROI detection model.

#### Landmark prediction

Because the first model of ROI detection was trained independently, separate datasets were generated for the second model. The second model, U-Net^11^ was used to find the exact locations of each landmark within the ROI patch obtained from the first model. In addition, two models with small ROIs (256, 256) and large ROIs (512, 512) were trained independently. The centre of ROI patches was represented as $\left( {\left| {T_{x} - D_{x} \left| {,{ }} \right|T_{y} - D_{y} } \right|} \right)$ instead of $\left( {T_{x} ,{ }T_{y} } \right)$ and the ROI detection’s mean distance error $\left( {D_{x} ,{ }D_{y} } \right)$ were extracted. The circular segmentation labels with the diameter $d$ were generated in the centre of ROI. If the diameter $d$ was too small, the information may be lost during CNN's training process. Conversely, the larger $d$ lead to the greater the prediction error of the model. Through several experiments, the most appropriate $d$ was empirically determined as 50 pixels.

Various augmentation methods such as Gaussian noise, random brightness, blurring, random contract, flip, and random rotation were used to train the segmentation model. Adam was used as a optimization function the learning rate was initially set to 0.0001, and then decreased by a factor of 10 when the validation set accuracy stopped improving in the two networks. In total, the learning rate was decreased 3 times to end the training. Dice similarity coefficient (DSC) was applied by calculating both the loss function and the model performance. For ablation study to evaluate the effectiveness of the first ROI detection, three models with and/or without ROI detection with fixed and variable ROI sizes were evaluated by using the average distance errors of all landmarks?

### Statistics analysis

The accuracy of ROI detection was evaluated by the distance between the predicted centres and ground truth ROIs. Statistical comparisons between models with the ROI detection only, without the ROI detection, and with the ROI detection using fixed size and variable size, were carried out to determine whether the model’s performances were significantly better. Paired t-test analyses with two-sided were performed for evaluating accuracy comparison of landmark prediction of the three models. The significant alpha was considered as 0.05 (p < 0.05) in this study. To compare the reproducibility of landmark prediction error of the cascade model and the expert orthodontist, a total of 28 cephalometric X-ray images from 28 patients was randomly selected and manually measured by the orthodontist with an interval of 6 months. Differences in the landmark's positions over the two trials were calculated as reproducibility, which was compared with those of the deep learning model. All statistical evaluations were performed by MEDCALC (MedCalc software, Ostend, Belgium) version 19.1.3 in this study.

## Results

### ROI detection

**Figure 4:**
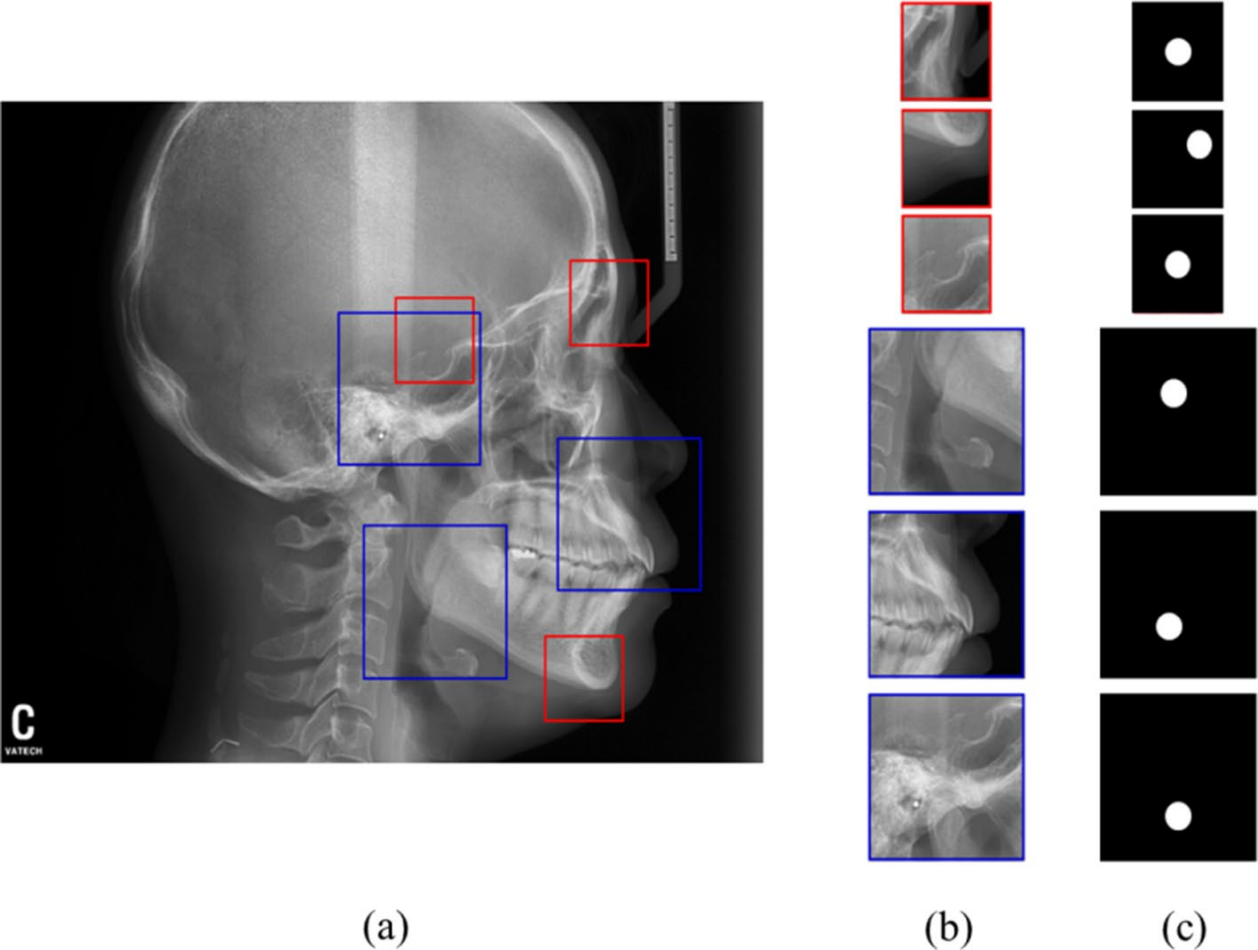
Regions of interests (ROIs) detection and landmark prediction results with different sizes depending on the information of each landmark. (**a**) Predicted ROIs (red and blue boxes) by RetinaNet algorithm, (**b**) ROI patches used for input of semantic segmentation for predicting a landmark, and (**c**) ground truth masks from the test set of each landmark.

Figure 4 shows the results of ROI detection and landmark prediction with different sizes depending on the required information of landmark prediction. Landmarks with small ROIs of 256 × 256 (red box) including the sella, nasion, and menton and large ROIs of 512 × 512 (blue boxes) including a-point, porion, and corpus left were predicted by RetinaNet algorithm. Based on these ROI regions, patches were extracted for input to semantic segmentation network, and U-Net for predicting a landmark. The mean and standard deviation of distance errors between the predicted centre of these ROIs and the ground truth of all the landmarks were 1.55 ± 2.17 mm (Table 2).

**Table 2:** Comparisons of distance error (mean ± STD, unit: mm) between predictions of the four different networks and the ground truth in the test set.

Methods	Mean ± STD	*p*-value^a^
Only ROI detection	1.55 ± 2.17	0.002
w/o ROI detection	1.92 ± 2.42	0.000
Mask R-CNN	3.59 ± 2.61	0.000
w/ ROI detection (segmentation/fixed size)	1.29 ± 1.39	0.003
w/ ROI detection (segmentation/variable size)	**0.79 ± 0.91**	**–**
w/ ROI detection (regression/variable size)	2.63 ± 1.13	0.000

*ROI* region of interest, *STD* standard deviation, *w/o ROI detection* landmark prediction without ROI detection, *Mask R-CNN* end-to-end model for ROI detection and landmark prediction with Mask R-CNN, *w/ ROI detection (segmentation/fixed size or segmentation/variable size or regression /variable size)* Landmark prediction with ROI detection of fixed or variable size.

^a^The accuracy of w/ROI detection (segmentation/variable size) was compared with the other accuracies with paired t-test.

### Landmark prediction

**Figure 5:**
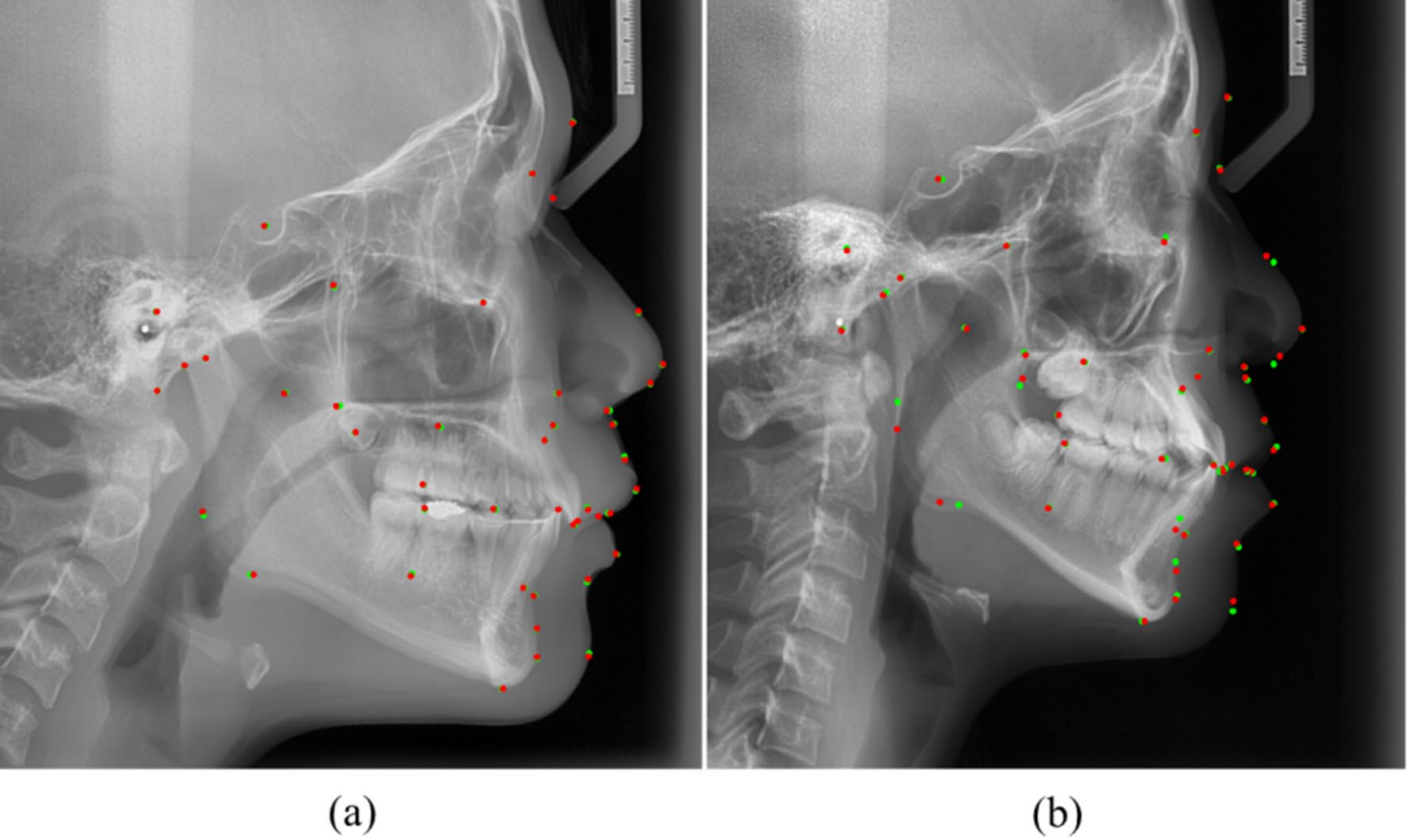
Experimental results of our proposed model. (**a**) shows the highest accuracy, and (**b**) shows the lowest accuracy in cephalometric X-ray images (red point, predicted landmark by deep learning; Green point, the ground truth).

The mean and standard deviation of the distance errors with or without the ROI detections of fixed and variable sizes experiments were listed in Table 2. The landmark prediction with ROI detection of variable size shows the best accuracy of all models (Table 3). In landmark-based analyses, each distance error of all landmarks predicted by the two models without ROI detection and with ROI detection of variable sizes was compared in Table 4. Approximately 55% of landmarks in prediction with ROI detection of variable sizes showed significantly better accuracies. To validate our model, we also conducted comparative experiments with the previous methods^15,22^. The proposed model shows significantly better performance than those of the previous models including Mask R-CNN. In addition, considering the various patch sizes and depth of U-Net, a U-Net model with variable patch size and 5 depth was selected based on experimental result (Fig. 5).

**Table 3:** Comparisons of distance error (mean ± STD, unit: mm) using ninefold cross validation.

k-fold	1	2	3	4	5	6	7	8	9
Mean ± STD	**0.79 ± 0.91**	0.85 ± 0.92	0.83 ± 0.90	0.79 ± 0.96	0.85 ± 0.93	0.83 ± 0.92	0.83 ± 0.91	0.79 ± 0.93	0.83 ± 0.96
*P*-value	**-**	0.643	0.755	1.000	0.645	0.757	0.756	1.000	0.762

ninefold cross-validation results. The accuracy of w/ ROI detection (segmentation/variable size) was compared with the other accuracies with independent t-test.

*STD* standard deviation.

**Table 4:** Comparisons of distance error (Mean ± STD, unit: mm) of each landmark between prediction without the ROI detection and with the ROI detection of variable size.

Index	w/o ROI	w/ ROI^a^ (fixed size)	w/ ROI^b^ (variable size)	Index	w/o ROI	w/ROI (fixed)	w/ROI (variable)
0	0.60 ± 0.34	0.38 ± 0.27	0.24 ± 0.32**	21	9.04 ± 2.11	0.48 ± 0.53	0.48 ± 0.53**
1	1.09 ± 0.63	1.12 ± 1.08	0.88 ± 1.06	22	1.70 ± 5.22	1.25 ± 1.15	1.25 ± 1.15
2	1.24 ± 0.79	0.87 ± 1.08	0.76 ± 1.06**	23	1.59 ± 1.00	2.31 ± 2.91	2.00 ± 2.64
3	1.14 ± 0.75	1.42 ± 1.99	1.22 ± 1.62	24	0.70 ± 0.40	0.69 ± 0.79	0.69 ± 0.79
4	1.06 ± 0.61	1.15 ± 1.36	1.00 ± 1.36	25	0.94 ± 0.57	1.12 ± 1.14	0.94 ± 0.85
5	0.85 ± 0.56	0.4 ± 0.41	0.38 ± 0.42**	26	0.80 ± 0.40	0.68 ± 0.64	0.68 ± 0.64
6	1.19 ± 0.62	0.92 ± 0.94	0.92 ± 0.94*	27	1.70 ± 4.90	2.03 ± 4.19	1.21 ± 2.44
7	1.25 ± 0.70	1.08 ± 1.13	1.08 ± 1.13	28	2.94 ± 10.06	1.71 ± 1.83	1.36 ± 1.48
8	1.22 ± 0.81	0.82 ± 0.79	0.67 ± 0.73**	29	1.20 ± 0.80	1.05 ± 1.15	1.01 ± 1.08
9	1.16 ± 0.68	0.64 ± 0.60	0.50 ± 0.58**	30	1.46 ± 3.25	0.60 ± 0.46	0.60 ± 0.46*
10	1.41 ± 1.03	1.24 ± 1.23	1.06 ± 1.07*	31	1.95 ± 5.71	0.97 ± 0.98	0.97 ± 0.98
11	0.91 ± 0.52	6.62 ± 7.22	0.92 ± 1.00	32	1.13 ± 0.56	0.55 ± 0.42	0.55 ± 0.42**
12	1.26 ± 0.81	0.68 ± 0.70	0.68 ± 0.70**	33	1.30 ± 0.83	0.46 ± 0.35	0.46 ± 0.35**
13	1.67 ± 3.05	8.18 ± 10.19	2.12 ± 2.38	34	1.41 ± 1.01	0.91 ± 1.03	0.85 ± 0.88**
14	1.14 ± 0.62	1.59 ± 1.78	1.44 ± 1.50	35	1.41 ± 0.98	0.88 ± 0.90	0.88 ± 0.90**
15	0.89 ± 0.58	0.72 ± 0.72	0.72 ± 0.72	36	0.98 ± 0.63	0.81 ± 0.70	0.74 ± 0.67*
16	19.08 ± 31.91	3.10 ± 6.40	1.76 ± 1.91**	37	1.04 ± 0.68	0.91 ± 0.81	0.78 ± 0.80*
17	3.15 ± 11.01	1.84 ± 2.05	1.63 ± 1.93	38	1.01 ± 0.66	0.71 ± 0.62	0.71 ± 0.62**
18	0.80 ± 0.59	0.73 ± 0.51	0.53 ± 0.47**	39	1.49 ± 1.03	0.98 ± 1.33	0.98 ± 1.33**
19	0.66 ± 0.36	0.40 ± 0.42	0.40 ± 0.42**	40	1.82 ± 1.64	1.96 ± 2.00	1.07 ± 1.20**
20	1.55 ± 0.82	1.30 ± 1.35	1.26 ± 1.30	41	2.05 ± 1.70	2.50 ± 3.49**	0.96 ± 1.21**

*ROI* region of interest, *STD* standard deviation, *w/o ROI* Landmark prediction without ROI detection, *w/ROI (fixed or variable)* landmark prediction with ROI detection of fixed or variable size.

*p-value < 0.05; **p-value < 0.005 using paired t-test.

^a^The accuracy of landmark prediction with ROI detection of fixed size was compared with the accuracies of those without ROI detection.

^b^The accuracy of landmark prediction with ROI detection of variable size was compared with the accuracies of those with fixed size.

To avoid overfitting, ninefold cross validation was conducted in Table 3.

### Comparison with reproducibility of an expert orthodontist

To measure the reproducibility of the landmark prediction error of an expert orthodontist, a total of 28 cephalometric X-ray images from 28 patients was randomly selected and manually measured by the orthodontist after 6 months. Differences in the landmark's positions in the two trials were calculated as reproducibility and were compared between the different models. The orthodontist had a mean reproducibility and standard deviation of distance error of a total of 42 landmarks of 0.80 ± 0.79 mm and mean reproducibility and standard deviation of distance error of landmarks were listed in Table 5, which shows considerably similar accuracies of landmark prediction with ROI detection of variable size.

**Table 5:** Comparisons of distance error (mean ± STD, unit: mm) between first label and second label. (unit: mm).

Index	Reproducibility distance error	Index	Reproducibility distance error
0	0.30 ± 0.21	21	0.43 ± 0.32
1	0.80 ± 0.67	22	0.67 ± 0.56
2	0.71 ± 0.91	23	0.90 ± 0.91
3	0.70 ± 0.60	24	0.65 ± 0.67
4	0.99 ± 1.07	25	0.89 ± 0.75
5	0.52 ± 0.58	26	0.56 ± 0.44
6	0.50 ± 0.44	27	1.10 ± 0.79
7	0.67 ± 0.65	28	1.32 ± 1.11
8	1.02 ± 0.91	29	0.87 ± 0.77
9	0.60 ± 0.68	30	0.53 ± 0.43
10	1.06 ± 1.25	31	0.87 ± 1.15
11	1.02 ± 1.16	32	0.36 ± 0.31
12	0.52 ± 0.60	33	0.53 ± 0.51
13	1.15 ± 1.14	34	0.70 ± 0.67
14	1.24 ± 1.12	35	0.57 ± 0.47
15	0.50 ± 0.53	36	0.54 ± 0.51
16	2.35 ± 2.15	37	0.70 ± 0.66
17	1.12 ± 1.21	38	0.42 ± 0.40
18	0.39 ± 0.41	39	0.98 ± 1.67
19	0.31 ± 0.28	40	1.55 ± 2.24
20	1.32 ± 0.23	41	0.90 ± 0.94

Landmarks measured for the first time in 28 X-rays were called first labels, and landmarks measured for the same patient after 6 months were called second labels.

## Discussion

Cephalometric x-ray images could provide orthodontists important information to determine orthodontics and maxillofacial surgery treatment options. However, the quality of cephalometric analysis depends on the accuracy of delineating landmarks, which could be vulnerable to inter- or intra-observer variations. In addition, the extensive number of landmarks requires that orthodontists spend considerable time per analysis for each patient, leading to fatigue. The present study introduces a new algorithm to increase CNN performance in cephalometric landmark identification in a fully automated manner.

The size of the original cephalometric x-ray images was too large, and irrelevant information could prevent from predicting landmark precisely with only one network. Therefore, in this study, we proposed a cascade CNN which consists of two steps to transfer learning manner. In the first step, the ROIs were predicted by using RetinaNet. In the second step, U-Net was used to detect the precise landmarks in those ROIs with relevant information, which significantly enhanced the overall accuracy of this landmark prediction to those of the other methods (Table 2). Furthermore, we demonstrated superior performance over recently existing regression-based models^22^ and single detection models^15^.

In general, orthodontists need a variable field of view to detect each landmark, which leads to training the model with variable sized ROIs. To identify the landmark, it was more effective to match the ROI sizes of each landmark to the field of view of the orthodontist. In addition, this method shows substantially better intra-observer variation compared to the orthodontist, meaning that this method shows robust accuracy.

Previous studies investigated a limited number (< 20) of hard tissue landmarks, and the results could not be satisfactory in clinical orthodontic practice^12,13^. Recently, Hwang et al. reported the accuracy of 42 landmarks, including 23 hard tissue landmarks and 19 soft tissue landmarks^16^. However, the study did not consider all possible landmarks for hard tissue analysis, soft tissue analysis, and occlusal plane analysis. With the results of this model, we could analyse the occlusal plane as well as hard and soft tissue analysis.

In this study, there are several limitations. First, this study was only evaluated with a dataset from a single centre and a single observer. Therefore, we need to extend this study with datasets from multi-centres, multi-vendors, and multi-observers. We suspected that the high quality of gold standard for training by as single observer would cause the accuracy of our model to be comparable to those of an expert orthodontist. In addition, this study could suffer from disease prevalence, partially caused by a single centre. Therefore, we need to test our model in varied clinical settings of maxilla-facial surgery and plastic surgery, which need to automated cephalometric analysis, as well.

## Conclusion

In this paper, we propose the idea of connecting two different models in a cascade manner to develop a fully automated landmark prediction model in cephalometric x-ray images. The model with the cascading CNN with variable ROI size shows significantly better accuracy than the other models, and is comparable to the expert orthodontist with more than 20 years’ experience and could be applied in actual clinical practice.

## Data Availability

This retrospective study was conducted according to the principles of the Declaration of Helsinki, and was performed in accordance with current scientific guidelines. The study protocol was approved by the Institutional Review Board Committee of Seoul National University School of Dentistry and Seoul National University Dental Hospital, Seoul, Korea (S-D 2018010 and ERI 19007). The requirement for informed patient consent was waived by the Institutional Review Board Committee of Seoul National University School of Dentistry and Seoul National University Dental Hospital.
